# Field emission performance of graphene-incorporated aluminum-based metal matrix composite†

**DOI:** 10.1039/d4na00646a

**Published:** 2024-11-25

**Authors:** Sunil Kumar Pradhan, Pandiyarajan K., Shubham Patil, Padmakar G. Chavan, Raphael Longuinhos Monteiro Lobato, Jenaina Ribeiro-Soares, Dattatray J. Late

**Affiliations:** a School of Electronics Engineering, Vellore Institute of Technology Chennai 600127 India sunilkumar.pradhan@vit.ac.in; b School of Advanced Sciences, Vellore Institute of Technology Chennai 600127 India; c Department of Physics, School of Physical Sciences, Kavayitri Bahinabai Chaudhari North Maharashtra University Jalgaon 425001 India; d Department of Physics, Federal University of Lavras, Campus Universitário PO Box 3037 Lavras Minas Gerais 37200-000 Brazil jenaina.soares@ufla.br datta099@gmail.com

## Abstract

The ‘close proximity’ configuration was used for field emission analysis of graphene-incorporated aluminum (Al) composites. The turn-on field was found to be 2 V μm^−1^ for the AlGr1 (1% graphene (by weight) inside the Al matrix) composite compared to 4.75 V μm^−1^ for the pristine aluminum sample. As the potential was increased, the current due to emission expeditiously increased in an electric field of 4 V μm^−1^, with 1 mA cm^−2^ current density due to emission for the AlGr1 composite, compared to that of 1.08 μA cm^−2^ for Al. The turn-on value was visually perceived to be superior for the AlGr1 composite as compared to the value for Al. Also, a quite stable emission current was recorded for the AlGr1 composite as compared to Al. Furthermore, the composites maintained approximately 98.7% of the density of pure aluminum following the sintering process. The structural wholeness and the nonexistent porous quality of the sintered specimens was confirmed *via* X-ray micro-computed tomography (micro-CT). The thermal amalgamation of the AlGr composite materials at 640 °C was found to be adequate, and produced composites with the desired strength. These evaluations indicate that AlGr composites can be excellently applied as cathodes and for the prevention of crumpling of electrical line cables.

## Abstract 2

The aluminum-incorporated graphene was used for the enhanced field emission performance.

## Graphical abstract

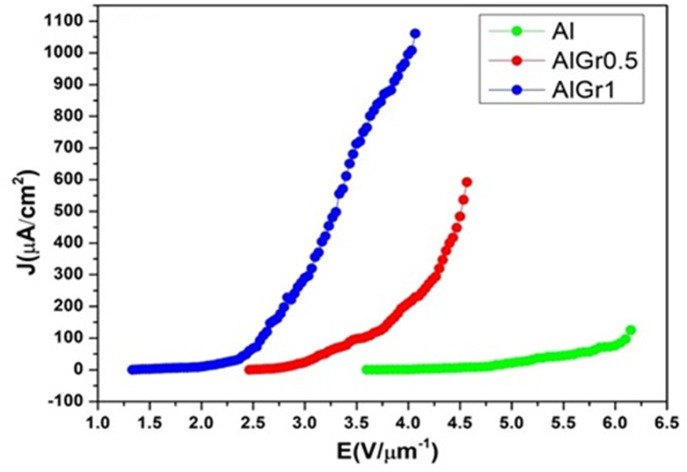

## 1. Introduction

There has been a broad range of technological implementation of metal matrix composites due to their outstanding thermal and mechanical properties. The significant capabilities of the thermo-electrical structures of metal matrix composites are utilized in nano- and micro-devices and heat collector materials.^1–4^ In the case of metal matrix composites, to achieve certain desired results, vigilant examination and alteration of various evaluative factors are indispensable. Important aspects such as volume fraction, percentage of weight, orientation, shape, and size can have considerable effects on the properties of the endmost composite sample.^5,6^

Another crucial feature is the development of the connections between the metal matrix and the dispersed phase, which can confer interesting features to the composite structure, and produce outstanding changes to the chemical and physical characteristics.^7,8^ The alignment of the incorporated entity in the metal matrix can be homogeneous or non-homogeneous, and it can be governed to accomplish the appropriate outcomes.

Aluminium matrix composites are currently being used in the construction of thermo-mechanical/thermo-electrical equipment and automobiles, and have been applied in the aerospace industry, and in a massive number of materials used to create infrastructure.^9–12^ The benefit of aluminium over other well-known metals is that it is readily available because of its excessive presence in the Earth's crust. Above all, there are various distinctive properties of aluminium, *e.g.*, low density, excellent thermal conductivity, malleability, ductility, and resistance due to corrosion. However, the disadvantages of aluminium and aluminium-based alloys include average mechanical strength and low wear resistance.^13^ Thus, numerous reinforcement techniques have been examined where small quantities of aluminium oxide (Al_2_O_3_), boron nitride (BN), or silicon carbide (SiC) have been incorporated into aluminium to form aluminium matrix composites with enhanced thermal, electrical, and mechanical properties.^14–18^

Carbon nanotubes (CNTs) and graphene have recently been used as ideal reinforcement materials for aluminium matrix composites because of their enhanced thermal, mechanical, electrical, ductile, and lubricating properties.^19–22^ Graphene is a popular carbon allotrope with outstanding physicochemical characteristics, *e.g.*, high electron mobility, excellent in-plane thermal and electrical conductivity, and excellent chemical and thermal stability.^23–25^ Graphene as an incorporated entity can remarkably enhance the inherent characteristics and effects of the metal matrix. An appreciable improvement in the transport properties of the metal can be accomplished by incorporating a minute weight percentage of graphene into the metal matrix system. The electrical and also the thermal transport at the boundaries between the metal and graphene is of paramount importance for the purpose of device and industrial applications.

To construct a layout of systematized thermo-mechanical/thermo-electrical energy devices, it is immensely imperative to determine the electrical and thermal transport behavior in the composite that occurs between the given metal and the graphene. It is also important to consider that the preparation of the graphene from the graphite is an economical procedure that permits mass production at the industrial scale. The synthesis of aluminium matrix composites with graphene as the incorporated entity can be carried out with various approaches that can be widely classified as the dispersion technique or the interfacial reaction procedure. Between these two, the dispersion technique permits homogeneous dispensation and preferable coalescence of the matrix and incorporated entity. The commonly implemented dispersion technique *via* powder metallurgy consists of high energy ball-milling (HEBM).^26^ The advantage of HEBM is that more homogeneous dispensation of the incorporated phase occurs.

In the present research work, graphene was employed as the incorporated entity, and aluminium was used as the metal matrix. An economical technique utilizing powder metallurgy was performed to acquire AlGr composites with a lowered value of the co-efficient of thermal expansion (CTE) in comparison to the pure aluminium.^26^ Thus, the composite could be applied to prevent crumpling of electrical line cables. Also, the composites showed encouraging results with respect to field emission performance for possible application as cathodes. Intriguingly, the composite perpetuates every physical characteristic of the aluminium component intact.

## 2. Experimental section

### Materials and methods

Aluminium powder (99.9% purity) and 2 to 5 layers of research grade graphene (>99.6% carbon) were procured from a commercial source (Platonic Nanotech Private Limited). Because aluminium is very reactive with oxygen, aluminium oxide (Al_3_O_3_) can form on the surface of aluminium (Al) powder. This oxide layer can cause problems in a variety of operations, especially those that require the preservation of aluminium's reactivity. To reduce or prevent aluminium oxide from forming on aluminium powder, we prevented exposure of the aluminum powder to oxygen by handling and storing it in an inert atmosphere, such as that under argon or nitrogen. Glove boxes or other sealed containers were used for this.

The oxide layer can also be reduced or eliminated using chemical or mechanical treatments. For example, the oxide layer can be partially dissolved by employing acid solutions (such as hydrochloric acid), although caution must be used to prevent harm to the aluminium powder. Controlling the milling environment (*e.g.*, by adding a milling agent or utilising a protective atmosphere) can assist in limiting the oxidation that occurs during milling while producing or processing aluminium powder. Also, to reduce exposure to air, aluminium powder can be stored in vacuum-sealed packaging or sealed airtight containers. The powder can also be kept in a dry, cool area to slow the oxidation process.

In our research, the powders were used as per their corresponding weight ratios in two separate zirconia jars accompanied by balls of zirconia in toluene. The proportion of balls to powder was sustained at the ratio of 20 : 1. With the assistance of a high-energy ball mill, the milling operation to prepare the aluminium–graphene (AlGr) composites was performed for 10 hours with 300 rpm (revolution per minute). The AlGr composites were formed into green pellets with the aid of a cylindrical die (length of 1.2 cm and diameter of 0.6 cm) and hydraulic press equipment. The load applied was 1.30 tons for three minutes during the course of the compaction. The green pellets were then vacuum-sintered at 640 °C for 1.5 hours, with a heating rate of 4 °C min^−1^. The thermal coalescence of the AlGr composites at 640 °C was observed to be adequate, and produced composites with the requisite strength.

## 3. Results and discussion

**Fig. 1:**
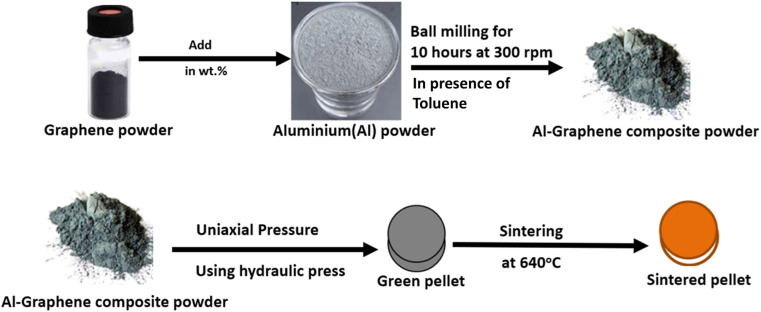
Schematic diagram illustrating the synthesis of aluminium–graphene (Al–Gr) composites employing high-energy ball milling (HEBM) and the use of vacuum sintering to fabricate the composite devices.

**Fig. 2:**
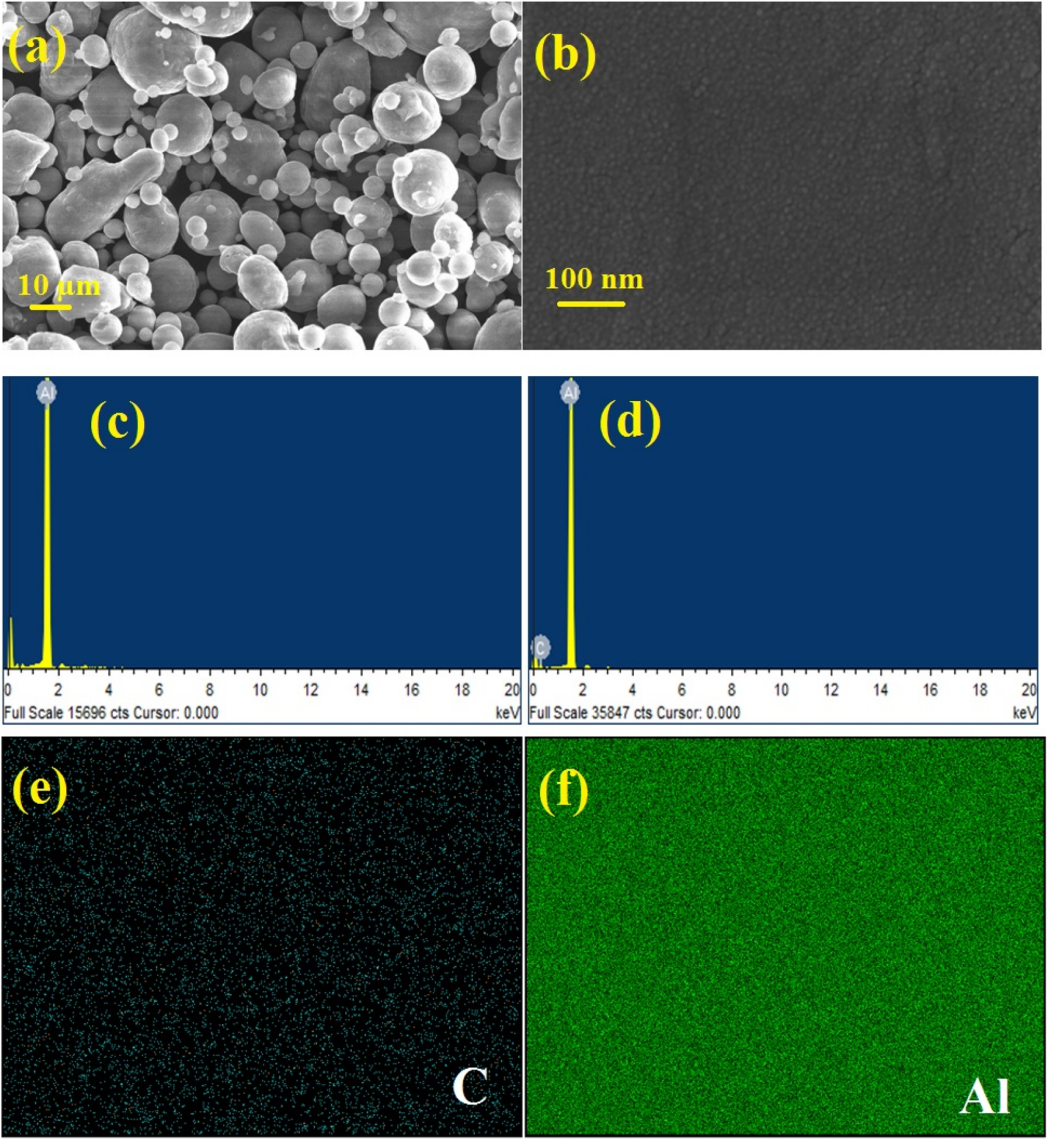
FE-SEM analysis of (a) aluminium powder and the (b) AlGr1 composite. The energy dispersive X-ray analysis (EDAX) for (c) pure aluminium, and the (d) AlGr1 sample. Electron mapping results representing the occupancy of (e) C and (f) Al and their dispensation in the AlGr1 composite.

In the present research work, pellets composed of aluminium and aluminium–graphene (AlGr) composites were examined. Different weight percentages (*i.e.*, 1, 0.75, 0.5, and 0.25) of graphene powder were introduced into the aluminium system. The synthesized aluminium–graphene composites were designated as AlGr0.25, AlGr0.5, AlGr0.75, and AlGr1. Fig. 1 shows a schematic illustration of the ball-milling process and the AlGr composite synthesis. Consequent qualitative and quantitative investigation *via* compositional and morphological analyses of the pure aluminium and the aluminium–graphene (AlGr) composites appear in Fig. 2(a–d). Fig. 2(a and b) display field emission scanning electron microscopy (FE-SEM) images of pure aluminium (Al) and AlGr1 composite (1 weight percent of graphene in the aluminium system), respectively.

The energy dispersive spectroscopy (EDS) analysis for pure Al and the AlGr1 composite are depicted in Fig. 2(c and d), respectively. As illustrated in Fig. 2(b), there is unvaried distribution of spherical nano-particles in AlGr1, with discrete sizes of the particles ranging from 15 to 20 nm, which enabled the unvaried coalescence of aluminium and graphene ingredients that is required for the AlGr composites to be utilized as cathodes, solar thermal collectors, and thermo-mechanical devices.

**Fig. 3:**
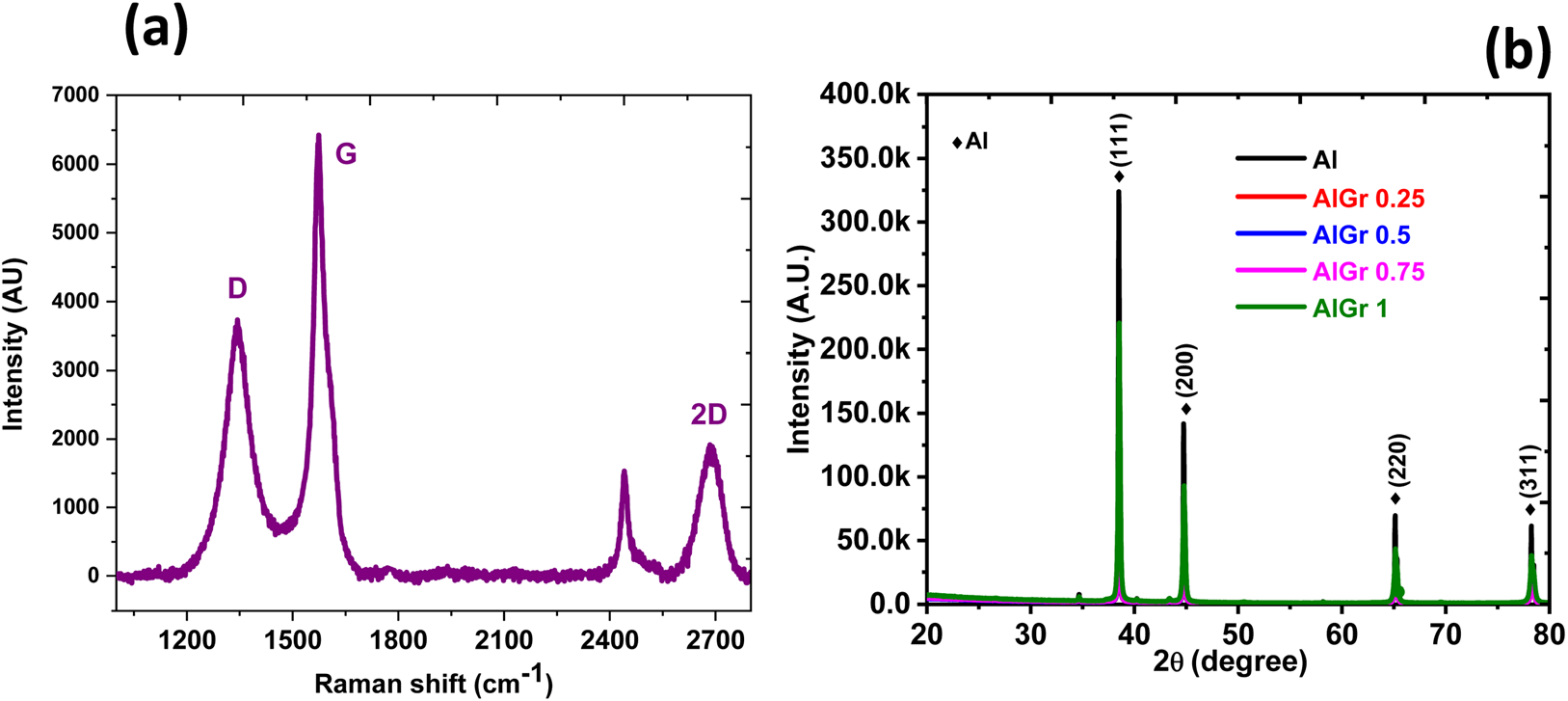
(a) Raman spectrum of graphene in the Al sample (AlGr). (b) XRD analysis of the AlGr composites.

Structural inspection of pure Al and all the AlGr composites (0.25, 0.5, 0.75, and 1 weight percent of graphene in the aluminium system) were carried out *via* X-ray diffraction (XRD), and the plots are shown in Fig. 3(b). The pristine aluminium and the AlGr samples (AlGr0.25, AlGr0.5, AlGr0.75, and AlGr1) exhibited similar growths of phase, *i.e.*, Miller indices planes, (311), (220), (200), and (111), that are the signature of the pure aluminium. However, the XRD intensities of all the samples are dissimilar, with variation from the extortionate for the pure aluminium to the modest for AlGr1. The non-existence of the prominent peak of graphene at approximately 27° for the (002) plane of the AlGr composite samples implies high crystalline characteristics of the aluminium or/and the incorporated graphene of a few 100 nm in thickness.

The Raman characterization showing signature graphene bands is exhibited in Fig. 3(a), and the D band at approximately 1295 cm^−1^ is muddled due to the edge effects, defects due to structural issues, and tumbling of sp^2^ hybridized carbon bonds. The G-band location is near 1575 cm^−1^, which is due to the in-plane oscillations of the sp^2^ hybridized carbon atoms. The presence of graphene in the AlGr sample was confirmed by the higher intensity peak of the G band in relation to the D-band. Fig. 3(a) also confirmed that the G and D bands are broad and not sharp, as is typically detected for pure graphene, and this can be due to the small amounts of graphene in the AlGr sample. The Archimedes principle was executed to evaluate the density of the sintered Al and AlGr composite samples. Following the sintering, the density of the aluminium–graphene (AlGr) composites was measured at approximately 98.7%.

The three-dimensional (3D) morphological features of AlGr1 were analyzed by micro computed X-ray tomography (Bruker, SkyScan 2211). This study validates the nonexistence of porosity of the sintered specimens along with the external and internal morphological features and micro-structural inspection. The restoration of the scanned results was accomplished *via* Sky Scan's InstaRecon software. The sintered specimen was scanned with 3000 rotations (0.18 increments) at an acceleration potential of 180 kV and a current of 220 μA. The restored results were shifted into a 3D configuration. Fig. S1(a and b)† illustrate the features of the surfaces and internal regions for the AlGr1 sintered specimen, respectively. The specimen was utilized in its original form, and Fig. S1† shows a 3D tomographic image of the sample. From the tomographic analysis, it is evidenced that our sintered AlGr1 sample is non-porous in nature, and the sample is free from any type of structural defects.

Field emission is a highly surface-sensitive phenomenon that depends upon various parameters of the emitter such as work function, electrical conductivity, surface topography, emitter size, and elemental composition. In the present case, graphene powder was added to aluminium powder to form an AlGr composite material. The field emission behaviour in terms of a turn-on field was observed to be 4.75 V μm^−1^, 2.8 V μm^−1^, and 2 V μm^−1^ for Al, AlGr0.5, and AlGr1 respectively, with superior performance observed for the AlGr1 nanocomposite. The observed field emission properties of AlGr1 may be due to the change in surface topography as well as the change in the elemental composition (due to nanocomposite formation).

The well-interconnected atoms of carbon in graphene in the form of an elongated sheet-like structure may be responsible for contributing to the easy transportation and percolation of electrons across the sample. This may also be possible because of the creation of additional protruding electron-emitting sites, which results in a low turn-on field. The field emission system for the samples was engaged for inspection of the current (*I*)–time (*t*) and field emission current density (*J*)–electric field (*E*) quantifications. Proper vacuum was maintained, and a pressure of approximately 3 × 10^−8^ mbar was conserved throughout the field emission analysis.

**Fig. 4:**
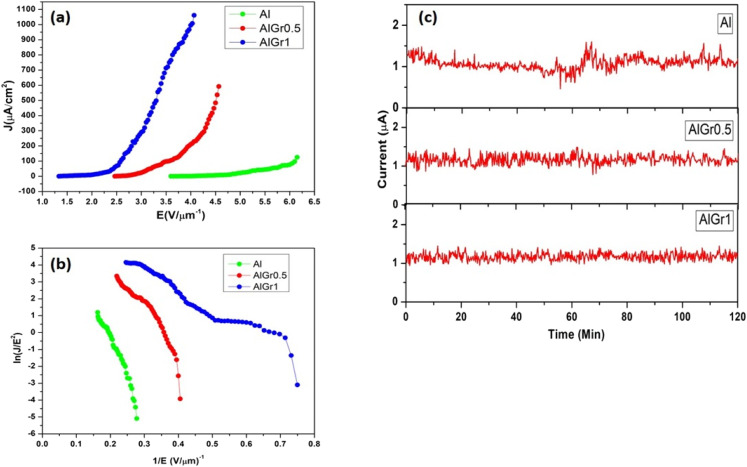
(a) Field emission current density (*J*)–applied field (*E*) and (b) Fowler–Nordheim (F–N) plots of Al, and the AlGr0.5 and AlGr1 nanocomposites. (c) Current (*I*) *versus* time (*t*) plot of Al, and the AlGr0.5 and AlGr1 nanocomposites recorded for the preset value of 1 μA.

The ‘close proximity’ configuration was performed for the field emission analysis, where aluminium, the aluminium–graphene composite containing 0.5 wt% of graphene (AlGr0.5), and the aluminium–graphene composite containing 1 wt% of graphene (AlGr1) served as the cathode. The cathode was fixed on the sample holder with the avail of conducting vacuum carbon tape. The anode-to-cathode gap was maintained at 0.1 cm. The field emission system was operated utilizing an ultra-high vacuum (UHV) system. The calibration of current was accomplished by estimating the voltage across the resistor. The area of all samples was 0.25 cm^2^. The applied electric field is defined as *E* = *V*/*d*, where *V* denotes the applied voltage, and *d* denotes the distance between the cathode and anode. The current densities due to field emission (*J*)–electric field (*E*) characteristics of the Al, AlGr0.5, and AlGr1 nanocomposites are shown in Fig. 4(a).

The turn-on field is defined as the field required to draw an emission current density of approximately 10 μA cm^−2^, and was found to be 4.75, 2.8, and 2 V μm^−1^ for the Al, AlGr0.5, and AlGr1 nanocomposites, respectively. As the potential was further increased, the current due to emission expeditiously increased, and the current density due to emission of 1.08 μA cm^−2^, 0.21 mA cm^−2^, and 1 mA cm^−2^ was drawn at an applied field of 4 V μm^−1^ for the Al, AlGr0.5, and AlGr1 nanocomposites, respectively. The turn-on numerical value was visually perceived to be superior for AlGr1 than the values for Al and AlGr0.5. The Fowler (F)–Nordheim (N) plots of Al, and the AlGr0.5 and AlGr1 nano-composites are depicted in Fig. 4(b), which shows their non-linear characteristics. As far as device fabrication is concerned, the reliability and constancy of the field emission current are significant factors along with the increased performance. The emission current (*I*) *versus* time (*t*) plot of Al, and the AlGr0.5 and AlGr1 composites are exhibited in Fig. 4(c).

The pre-set value of the emission current at 1 μA was considered for inspection of the current (*I*) *versus* time (*t*) plot for a time span of 2 hours. Over a period of 2 hours of testing, very stable emission current was indicated for Al, and the AlGr0.5 and AlGr1 nanocomposites. Compared to AlGr1, we perceived slightly higher instabilities in Al and AlGr0.5, with slightly higher instabilities in Al compared to AlGr0.5. The turn-on field value for AlGr1 was quite superior as compared to the other reported composites of Al and graphene. Table 1^27–32^ summarizes a comparison of turn-on fields. Because there are no reports on field emission studies of aluminium–graphene composites, the comparison was performed with other composites of Al and graphene. Electron emission stability is one of the decisive parameters for practical applications of electron emitters.^33–38^

**Table 1:** Comparison of the turn-on field between the aluminum–graphene composite and reported composites of aluminum and graphene

Specimen	Turn-on field V μm^−1^ at *J* = 10 μA cm^−2^	References
Graphene sheets	4.5	27
Graphene/ZnO hybrid nanorods	2.9	27
ZnO/graphene nanocomposite	2.1 (*J* = 1 μA cm^−2^)	28
MnO_2_/rGO nanocomposite	3.6 (*J* = 1 μA cm^−2^)	29
SnO_2_/graphene nanocomposite	3.85 (*J* = 1 μA cm^−2^)	30
AIN nanotips grown on Si	6	31
AlN nanocrystal	15.1 (*I* = 0.034 μA)	32
Al nanopowder	4.75	Present work
Aluminium/graphene composite (AlGr0.5)	2.8	
Aluminium/graphene composite (AlGr1)	2	

In the present study, the emission current stability was recorded for the preset value of 1 μA emission current for the duration of 2 h. The emission current stability of AlGr1 was found to be greater than that for AlGr0.5 and Al. The instabilities in emission current decreased as we went from Al to the AlGr1 sample during the testing, and the greater current stability was likely due to the robust nature of the AlGr1 sample. During field emission, the phenomenon of ion bombardment on the emitter surface was dominant, which affected the emitter morphology and further resulted in an increase/decrease of emission current (a sharp emitter becomes blunt or a blunt emitter becomes sharp due to ion bombardment).^33–38^ In the case of AlGr1, the greater stability and increase in the robust nature of the electron-emitting sites may be responsible for the enhanced emission stability. The small number of instabilities observed in the AlGr1 sample may be due to the phenomenon of adsorption and desorption of residual gas molecules on the emitter surface. Fig. S2† depicts the field emission mechanisms of the Al–graphene matrix.

## 4. Conclusions

We prepared aluminum–graphene composites employing a powder metallurgy technique, and an analysis of their field emission was performed. The composites showed unvaried distribution of spherical nano-particles, with discrete sizes of the particles ranging from 15 to 20 nm, which enabled the unvaried coalescence of the aluminum and graphene ingredients. From the field emission current density–applied electric field (*J*–*E*) characteristics, the turn-on field was recorded at 2 V μm^−1^ for AlGr1 compared to 4.75 V μm^−1^ for pristine Al to draw an emission current density of approximately 10 μA cm^−2^. As the applied voltage was increased, it was found that the emission current very expeditiously increased, and an emission current density of 1 mA cm^−2^ was drawn for the AlGr1 composite, compared to the emission current density of 1.08 μA cm^−2^ for Al at an applied field of 4 V μm^−1^. The turn-on value was visually perceived to be superior for the AlGr1 composite as compared to the value for Al. Also, a quite stable emission current was recorded for the AlGr1 composite compared to that for Al. Hence, from the field emission performance results, it can be concluded that the aluminum–graphene (AlGr) composite materials can be used for the potential application as cathodes in field emission-based devices.

## Data availability

All data generated or analyzed during this study are included in this published article and its ESI.†

## Author contributions

SKP, PK, SP, and PGC performed the synthesis experiments, characterizations, and analysis. The FE analysis was performed by PG, and DJL wrote the first draft of the paper. RLML, JRS, and DJL revised the writing of the manuscript and participated in data curation. DJL and JRS guided the research and conceived the project.

## Conflicts of interest

The authors declare no conflicts of interest.

## Supplementary Material

NA-OLF-D4NA00646A-s001
