# Supplementary material for: Field emission performance of graphene-incorporated aluminum-based metal matrix composite
Source: Nanoscale Adv. 2024 Nov 25;7(2):614–20. doi: 10.1039/d4na00646a (PMC11626629; doi:10.1039/d4na00646a)
Supplement: NA-007-D4NA00646A-s001 [file NA-007-D4NA00646A-s001.pdf]

Supplementary Information (SI) for Nanoscale Advances.
This journal is © The Royal Society of Chemistry 2024

Supplementary Material

Field emission performance of graphene incorporated aluminum based metal matrix composite

Sunil Kumar Pradhan[a]*, Pandiyarajan K[b], Shubham Patil[c], Padmakar G. Chavan[c], Raphael Longuinhos Monteiro Lobato[d], Jenaina Ribeiro-Soares[d]*, Dattatray J. Late[d]*

[a]School of Electronics Engineering, Vellore Institute of Technology, Chennai-600127 India
[b] School of Advanced Sciences, Vellore Institute of Technology, Chennai 600127, India
[c]Department of Physics, School of Physical Sciences, Kavayitri Bahinabai Chaudhari North Maharashtra University, Jalgaon 425001, India
[d]Departamento de Física, Universidade Federal de Lavras, Campus Universitário, PO Box 3037, Lavras, Minas Gerais 37200-000, Brazil

Figure S1

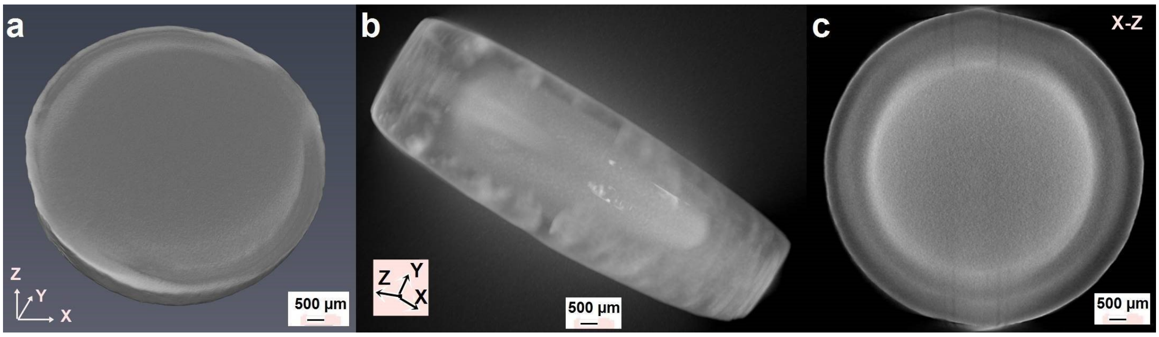

Figure S1: X-ray computed micro tomography (micro-CT) examination of aluminium-graphene sintered composite (AlGr1) (a) surface tomographic image (b) internal tomographic image (c) image of tomographic slice taken through centre of the specimen

Figure S2

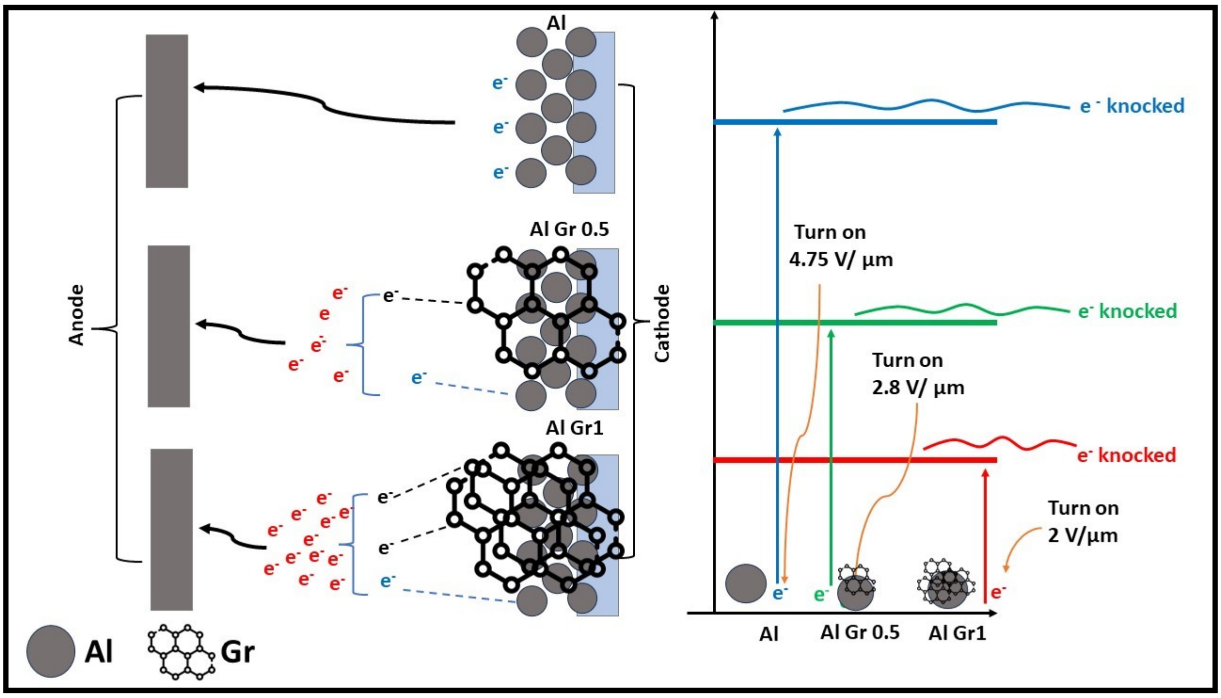

Figure S2: Field emission mechanisms of Al-graphene matrix.
